# RELATIONSHIP QUALITY AND OLDER PARENTS' COGNITIVE TRAJECTORIES: A NATIONAL LONGITUDINAL STUDY IN THE UNITED STATES

**DOI:** 10.1093/geroni/igac059.466

**Published:** 2022-12-20

**Authors:** Yan Zhang, Hui Liu

**Affiliations:** University of Wisconsin-Madison, Madison, Wisconsin, United States; Michigan State University, East Lansing, Michigan, United States

## Abstract

This study aims to assess the impact of the relationship quality between parents and children on parents’ cognitive function in later life, with an additional focus on variation by parents’ gender. We analyze data from a nationally representative longitudinal panel survey of participants age 50 and older. We employ latent growth curve models (LGCM) to estimate how changes in parent-child relationship quality are related to cognitive trajectories over time. Maintaining frequent contact with children and receiving more support from children are associated with a slower rate of cognitive decline for older parents whereas experiencing relationship strain with children is associated with a faster rate of cognitive decline for older parents. These associations are stronger for mothers than fathers. This study highlights the importance of the “linked lives” of aging parents and their children. The findings have implications for the development of interventions and strategies to protect cognitive function in later life.

